# Noninvasive Acoustic Recognition of Water Flow Sources for Human Activity Monitoring in Smart Homes

**DOI:** 10.3390/s25196221

**Published:** 2025-10-08

**Authors:** Sara Comai, Michele Cortinovis, Riccardo Girelli, Fabio Salice

**Affiliations:** 1 Department of Electronics, Information and Bioengineering (DEIB), Politecnico di Milano, 20133 Milan, Italy; sara.comai@polimi.it; 2 Department of Physics, Politecnico di Milano, 20133 Milan, Italy

**Keywords:** water flow recognition, human activity recognition (HAR), smart home monitoring, acoustic sensing, spectrogram analysis, activities of daily living (ADL), digital health

## Abstract

This paper presents a noninvasive system for identifying water flow sources with the final goal of supporting human activity recognition (HAR) in activities of daily living (ADL). The system employs a single microphone to capture ambient sounds within a room and detects active water sources based on their acoustic signatures. The audio signals are converted into time-resolved spectrograms, which are processed via time- and frequency-domain convolution and subsequently classified using a neural network. This approach enables both the identification of specific water sources, even combined, and the measurement of their usage duration with an overall accuracy of 90.6%. The study focuses on four common bathroom fixtures: toilet, bidet, shower, and washbasin. The proposed system is adaptable to various environments, requires no modifications to plumbing infrastructure, making it suitable for smart home and digital health applications.

## 1. Introduction

Monitoring water usage offers multiple benefits, among which the best known is water consumption management [1,2]. However, when integrated into a smart-household framework, this form of control supports multiple use cases that can positively impact people’s quality of life.

An important use case in this domain is the unobtrusive monitoring of elderly individuals living independently at home. Despite notable advancements in Human Activity Recognition (HAR), several challenges remain, particularly in tracking individuals during their Activities of Daily Living (ADL). Meanwhile, the global elderly population continues to grow rapidly. According to the World Health Organisation, there are currently around one billion elderly individuals worldwide; this number is expected to rise to approximately 2.1 billion by 2050, at which point older adults will outnumber younger people and constitute nearly 22% of the global population [3]. Ageing is often associated with disorganized behavior, cognitive decline, and memory impairments, which limit a person’s ability to carry out their daily routine tasks independently. Indeed, one of the most prevalent conditions affecting older adults is dementia (including Alzheimer’s disease), which is characterized, among other symptoms, by a prolongation of passive or complex activities such as showering or brushing one’s teeth [4,5]. Therefore, being able to monitor changes in the time spent on such daily activities may be valuable for detecting the early stages of the disease or evaluating its progression.

Monitoring water usage is important for many other reasons, including conserving freshwater, reducing energy waste, and informing better resource management [6,7].

Currently, there is a clear absence in the market of embedded systems that can accurately identify and estimate the sources of water flow, such as bathroom fixtures, basins, showers, and bathtubs, in a smart home environment. In most households, the only available information is the total water consumption measured by the main water meter, with no additional data on specific points of use. An effective solution should not only provide detailed source-level information but also offer noninvasive installation, requiring no professional plumbing work, and be low-cost to ensure accessibility for a broad range of users.

The purpose of the proposed system is to accurately recognize a specific water source and estimate its flow rate using only a microphone. The system is intended to be placed within a room, sufficiently close to the target water source to ensure optimal accuracy. Importantly, the proposed approach is entirely noninvasive and does not require any modifications or additions to existing plumbing installations. This paper investigates the feasibility of distinguishing between different water sources based solely on their acoustic signatures, with evaluation performed through the generation and visualization of spectrograms.

Although the long-term goal of this research is to support human activity monitoring for the early detection of dementia in elderly people, the current study represents a first step, focusing on the accurate identification of individual water sources using a fully noninvasive approach. Future work will build on this foundation to analyze water usage patterns and, more broadly, capture human activities within the household.

The paper is organized as follows. Section 2 provides an overview of related work. Section 3 outlines the proposed methodology for water flow analysis, including data acquisition, transformation, and source classification using a dense neural network. It also details the system architecture. Section 4 presents the experimental setup, including data collection, spectrogram analysis, neural network design, and classification results. Section 5 examines the impact of Min–Max normalization, while Section 6 discusses class balancing strategies. Section 7 describes the training and validation procedures, and Section 8 reports the test performance. Section 9 evaluates the system using synthetic data. Section 10 presents limitations of the study, while Section 11 discusses RAM requirements. Finally, Section 12 concludes the study.

## 2. Related Works

Currently, a wide range of professional flow meters is available on the market. However, most of these devices are designed primarily for industrial applications or scientific research, such as clinical diagnostics, soil monitoring, and other specialized domains. In such contexts, they are used to measure various fluid properties such as density, temperature, pressure, viscosity, etc. As a result, their cost and complexity make them unsuitable for our intended application in a smart home environment. Moreover, such a high level of precision is not required to extract meaningful information for Human Activity Recognition (HAR) and Activities of Daily Living (ADL) analysis. Some alternative solutions have already been developed for similar objectives, and the following paragraphs provide a brief overview of these approaches.

Froelich et al. [8] proposed HydroSense, a low-cost single-point pressure system capable of identifying which fixture has been used and estimating the corresponding water usage. It can be installed at any location within a home’s existing water infrastructure (e.g., an exterior hose bib, utility sink spigot, or water heater drain valve). It measures pressure changes and estimates the combined effects of Poiseuille’s Law parameters by sampling flow rates at strategic locations. It detects open/close events using a hierarchical classifier with an accuracy of 97.9% and estimates water usage with error rates comparable to those reported in empirical studies of conventional water meters. The proposed approach may be affected by water pressure fluctuation and changes in environmental factors (e.g., temperature and selected location). Moreover, their analysis focused on identifying fixture events in isolation; multiple simultaneous flows have not been analyzed.

Vafeas et al. [9] introduce a prototype based on piezoelectric elements to sense the vibrations of the pipes near the hot and cold-water taps. The system employs Support Vector Machines (SVMs) to identify four classes: no flow, hot flow, cold flow, and mixed flow. The classifier achieves an overall accuracy of 96.07%, with most misclassifications occurring between mixed flow and cold or hot flow. Calibration for each deployment is performed by turning on the tap and assigning the corresponding label.

Pirow et al. [10] adopt a nonintrusive approach by combining temperature sensors and an accelerometer to monitor hot water usage. The proposed system leverages vibration signals and temperature variations to detect hot water events and estimate both flow rates and consumption volumes without the need for direct plumbing modifications. They obtained an accuracy of 89% for flow rate estimation and 93% for volumetric usage estimation.

Gerka et al. [11] employed a piezoelectric sensor affixed to the pipe using adhesive tape, while Lannes et al. [12] integrated a similar sensor directly into the pipe wall during the manufacturing process, making it an integral component of the infrastructure. In both approaches, one sensor is required per pipe to be monitored. Water flow is inferred by detecting pressure fluctuations or turbulent flow. Both systems demonstrated promising performance in their respective evaluations.

Also, Fogarty et al. [13] propose a device that must be installed in the home’s existing infrastructure, providing good results with some specified and limited activities.

Forster et al. [14] installed thermistor sensors on both the hot and cold-water feed lines of a residential sink. Voltage readings from the thermistors were converted to temperature values and filtered using a 1-s moving average to reduce noise. Flow start and end times were detected by identifying temperature changes exceeding ±0.1 °C from stable room temperature, with rising or falling trends indicating hot or cold-water flow, respectively. The method correctly detected flow start and end events, but showed large errors in measuring water use duration. A key limitation is that short, long, or repeated water use can prevent the pipe from returning to room temperature, affecting accurate flow detection.

Marin-Garcìa et al. [15] propose the usage of CNNs on data collected from temperature, humidity, and CO_2_ sensors, to classify some typical activities. They obtained an accuracy of between 70 and 80% to automatically distinguish the main activities (toileting and showering) and lower accuracies for the activity of urinating, confusing it with hand washing and brushing teeth.

Noninvasive microphone-based approaches can be broadly classified into two methodological categories: capturing acoustic signals directly from or in proximity to water usage sources, and analyzing ambient sound patterns within the surrounding environment.

Hori et al. [16] propose a system that uses microphones to capture water pipe vibrations, transmitting noise-reduced data via low-power Thread mesh communication to a home server (Raspberry Pi 4, Raspberry Pi Ltd., Cambridge, UK). Machine learning algorithms (Support Vector Machine—SVM—and Random Forest) are applied to estimate the activity patterns of elderly users. Their results show that combining Random Forest with a simple moving average yields the highest accuracy in estimating water usage from sound pressure data. In particular, they achieved a recall rate of 75% in recognizing key activities such as washing dishes and cooking in the kitchen, and hand washing and tooth brushing in the washroom.

Chen et al. [17] presented an automated system for monitoring bathroom activities using acoustic analysis, recognizing sounds such as showering, flushing, and faucet usage. The system relies on carefully designed Hidden Markov Models (HMMs) combined with MFCC features to classify events. While experiments in both controlled and real-life settings showed encouraging results, achieving over 84% accuracy for most sound categories, HMMs are limited in handling signals with complex non-linear characteristics, such as water sounds from different sources in environments with varying acoustic properties. This limitation may affect the system’s robustness and generalizability across diverse indoor settings.

Hou et al. [18] developed a low-power microsystem that accurately recognizes water flow sounds within a wireless acoustic sensor network. Using a CNN-based (Convolutional Neural Network) sound event recognition model, the system can detect water flow sounds above 55 dB with an average accuracy of over 92.5%. Compared to existing nonintrusive approaches [19], acoustic sensing offers an alternative without line-of-sight or lighting constraints.

Hyun et al. [20] proposed a method to detect three bathroom activities (showering, flushing, and faucet usage) from water sounds, using a two-stage network based on W-YAMNet (Water-YAMNet) with transfer learning. In W-YAMNet, YAMNet’s output layer was replaced with another hidden layer containing 512 nodes, and an output layer with three nodes corresponding to SH, FL, and FA was added. While showing promising results on 10-min audio segments, the model is computationally heavy, requires a platform such as a Raspberry Pi 4 (Cortex-A72, 64-bit SoC @ 1.5 GHz, 4 GB), and presents challenges for deployment on more resource-constrained microcontroller systems, making implementation on low-power devices difficult.

Despite their good accuracy, most of these systems are costly, require calibration, precise positioning, or invasive installation, and are thus less suitable for widespread deployment in smart home environments.

Compared to existing approaches in the literature, our proposed method and system allow for the reliable identification of individual water fixtures even in the presence of overlapping sources and ambient noise from everyday activities, without requiring a calibration phase or precise sensor placement.

Our previous work [7] investigated the use of environmental microphones to detect water flow noise through noninvasive methods. Two techniques were tested: one based on spectrum analysis, and the other on spectrogram analysis combined with an LSTM (Long Short-Term Memory) model for fixture usage classification. The latter showed more promising results and served as a preliminary study for the current approach adopted in this work, which adopts a different method to improve robustness.

Despite their good accuracy, most of these systems are costly, require calibration, precise positioning, or invasive installation, which limits their suitability for widespread deployment in smart home environments. In contrast, our proposed method and system operate on a lightweight microcontroller platform, applying simple transformations to the 512-point FFT of the audio signal to eliminate calibration requirements, particularly those related to microphone-to-source distance, and to reduce the impact of both environmental noise and the natural variability of water flow sounds. These transformations (temporal and frequency convolutions and min–max normalization) enable the use of a dense neural network for classification, fully implemented on the microcontroller. As a result, the system reliably identifies individual fixtures even in the presence of overlapping sources and ambient noise, while transmitting only the classification outcomes via Wi-Fi. This significantly reduces bandwidth usage and better safeguards privacy compared to alternative approaches.

## 3. Methodology

The water flow source identifier system is designed to recognize the use of one or multiple water sources within a room (e.g., a bathroom) in a nonintrusive manner, without requiring any modifications to the existing plumbing systems. By identifying the specific source and recording usage times, this system enables effective monitoring and analysis of human activities. In particular, it facilitates a detailed examination of Activities of Daily Living (ADL) related to personal hygiene, offering valuable insights while preserving user privacy and minimizing any disruption.

### 3.1. Data Flow

The system must be positioned at a point in the room where it can reliably sense all the designated water sources with the required sensitivity. Furthermore, it should be placed away from potential sources of interference, such as pipes, fans, or heaters, as these may degrade signal quality by introducing noise within the same frequency range as certain water sources.

**Figure 1:**
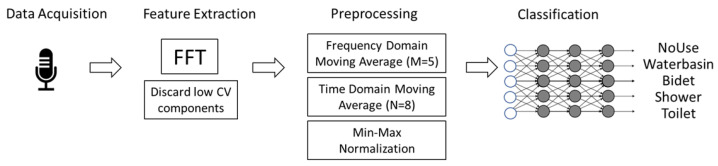
Overview of the main steps of the proposed pipeline, from raw audio acquisition to the final classification of active water sources, with intermediate steps.

**Figure 2:**
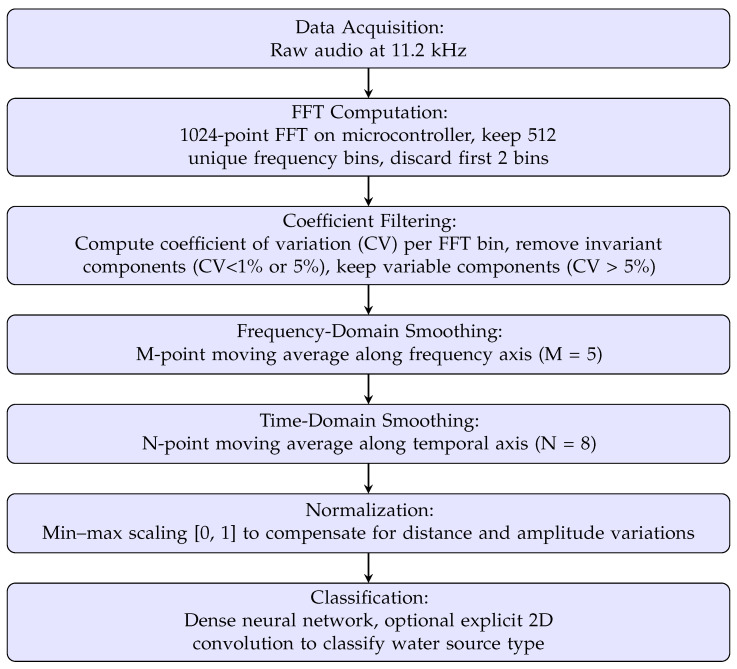
Detailed overview of the data acquisition and processing pipeline for water source identification.

The collected data are processed locally. Figure 1 provides an overview of the main steps of the processing pipeline, while Figure 2 summarizes the data processing pipeline, illustrating the steps from audio signal acquisition to classification of active water sources. After data acquisition, a continuous sequence of 512-point FFTs (Fast Fourier Transforms) is applied to extract the frequency characteristics of the noise captured by the microphone. The first two FFT components—including the DC component—are discarded; the numerical rationale for this choice is discussed in the Section 4.

From a methodological point of view, all FFT components with a coefficient of variation (CV—the ratio of the standard deviation to the mean) lower than 5%, calculated over the entire set of measurements for each scenario (i.e., same room type and microphone position), can be considered highly stable and invariant under the experimental conditions. As such, their contribution to source identification is essentially negligible. This implies that low-CV components are independent of the environmental conditions, meaning their values remain stable regardless of whether a tap, a shower, or other water sources are used.

Some FFT components exhibit higher sensitivity to experimental conditions and are therefore useful for distinguishing different water usage scenarios. Components with a coefficient of variation (CV) below 1% are considered invariant and are excluded from the model, whereas components with a CV above 5–10% provide relevant features for classification.

A two-stage filtering process is applied to the selected components. First, an M-point moving average in the frequency domain (M = 5) reduces fluctuations between adjacent frequency bins and enhances robustness to noise. Then, each frequency component, treated as a time series, is smoothed along the temporal axis with an N-point moving average (N = 8), which acts as a low-pass filter and further reduces residual noise.

The window sizes (M = 5, N = 8) were determined empirically to balance noise suppression and responsiveness to changes in water usage. This dual-stage filtering allows the classification to be performed using a simple dense neural network, without the need for more complex architectures such as CNNs, GRUs, or LSTMs.

Since the intensity of the acquired signal depends on the distance from the source, the doubly filtered FFT components are normalized using min–max scaling:

(1) $$\mathrm{Normalized}\;\mathrm{Value}=\frac{\mathrm{value}-\min}{\max-\min}$$

This normalization has a twofold effect: on the one hand, it makes source identification independent of the distance from the microphone; on the other hand, it compensates for amplitude variations across different recordings. Finally, it also enhances the effectiveness of the subsequent stage: the training of a dense neural network. Normalization of inputs, specifically min–max scaling in the [0, 1] range, provides several benefits for neural network training. First, it improves numerical stability and accelerates the convergence of the learning algorithm by keeping input values within a compact and consistent range, which aligns well with the operating domains of common activation functions. Second, it prevents certain input components with larger numerical values from dominating the optimization process at the expense of potentially more relevant features with smaller magnitudes. Additionally, normalization enhances the model’s ability to generalize by encouraging the learning of structural relationships among variables rather than spurious dependencies tied to their scale. Finally, using normalized inputs ensures better compatibility with widely adopted optimization algorithms such as SGD (Stochastic Gradient Descent), Adam (Adaptive Moment Estimation), and RMSprop (Root Mean Square Propagation, which perform more effectively when the input distributions are balanced and homogeneous. The rationale behind this choice lies in the fact that the relevant temporal features of the signal are effectively captured by the moving average, which also serves to smooth out variations caused by both measurement noise and environmental factors. As a result, the temporal dynamics are already represented in the preprocessed signal. The use of a double moving average, one in the frequency domain and one in the time domain, is an alternative to the use of convolutional neural networks (CNNs). In particular, the moving average applied over time to the FFT components, from $FFT_{i}(t-n)$ to $FFT_{i}(t)$, is formally interpreted as a discrete convolution operation between the time series x(n) and a uniform window. Specifically, given a window of width *M*, the moving average at a point *n* is computed as:

(2) $$y(n)=\frac{1}{M}\sum_{k=0}^{M-1}x(n-k)$$

This expression is equivalent to a discrete convolution

(3) y(n)=(x×h)(n).

where $h\left[k\right]=\frac{1}{M}$ per k=0,1,…,M−1, and h[k]=0 elsewhere.

In this way, the moving average operation can be seen as the weighted summation and overlap of the series values, progressively shifting the window along the time axis.

To complete the processing pipeline, classification is performed. The decision to employ a dense neural network combined with an explicit 2D convolution, rather than a full convolutional neural network (CNN), is motivated by the goal of implementing the model on resource-constrained microcontrollers and by the need for greater interpretability of the patterns observed in spectrogram representations. By explicitly applying the 2D convolution, this processing stage can be isolated, avoiding complete reliance on the automatic feature extraction mechanisms of CNNs. This approach reduces model complexity, limits the number of parameters compared to CNNs with multiple filters and layers, and facilitates a more transparent analysis of the extracted features. As a result, the model is more suitable for deployment in embedded systems and allows for clearer interpretation of spatial and temporal patterns relevant to the application.

### 3.2. Neural Network

Active source identification is achieved by training a neural network with as many input nodes as the number of informative FFT components. The network architecture consists of 100 nodes in the first hidden layer, 20 in the second, and 5 output neurons. ReLU activation functions are used for the hidden layers, while a sigmoid function is applied at the output layer to allow for multi-label classification. The test vectors refer to a possible scenario composed of $2^{N}-1$ distinct experiments, where *N* is the total number of water sources. In the case study, N=4 (toilet, bidet, shower, and washbasin), resulting in 15 scenarios representing all possible non-null binary combinations of active sources (from 0001 to 1111, where a 1 indicates an active source). An additional scenario representing background noise (i.e., all sources inactive) is also included. The test vectors consist of filtered FFT components (as described in the previous section, with moving average smoothing applied first in the frequency domain and then over time), and *N* binary output labels, each set to 1 if the corresponding source is active, and 0 otherwise. To avoid training bias, the number of vectors per scenario is kept constant.

The pre-trained network is used to identify the active water sources.

### 3.3. System Architecture

**Figure 3:**
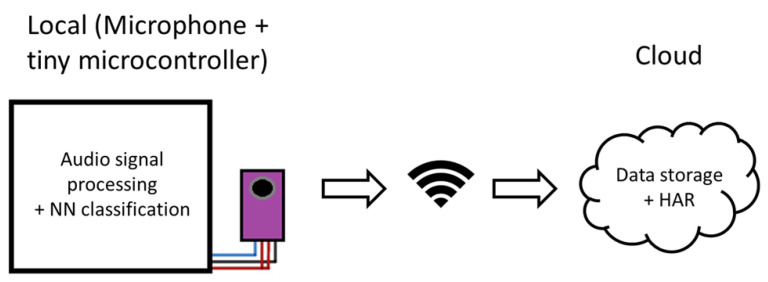
General system architecture. Audio is captured by a microphone and processed on a microcontroller. Classification is performed on-device, and results are sent to the cloud for storage and higher-level activity recognition.

The general architecture of the system is illustrated in Figure 3. Environmental audio is captured in the bathroom by a microphone (Electret Microphone Amplifier MAX9814, Adafruit Industries LLC, New York, NY, USA), and the raw signals are processed locally on a resource-constrained microcontroller (Adafruit Industries LLC, New York, NY, USA). A 1024-point FFT is computed on the device, producing 512 unique frequency bins. Approximately five FFTs are processed per second in the case study. Local processing also includes smoothing in both the frequency and time domains, as well as min–max normalization. Source classification is performed directly on the microcontroller using a pre-trained dense neural network optimized for low computational resources. This setup allows the classification to occur entirely on the embedded device.

The classification results, together with the feature vectors, are transmitted to the cloud for storage. In the cloud, human activity recognition is carried out by analyzing classified water usage events to infer higher-level behavioral patterns.

## 4. Experimental Results

A prototype was developed to evaluate the proposed method for identifying water usage sources. The acquired data were used both to configure the system (such as training the neural network) and to validate the method, by assessing its generality, limitations, and performance metrics, including precision and accuracy.

### 4.1. Experimental Setup

Experiments were conducted in different types of bathrooms in two apartments; in each type, the microphone was placed in a central position, but away from any pipes and specific water usage sources. All fixtures across bathrooms (washbasin, shower tray, bidet) were similar and all made of ceramic. The analyzed WC model was a toilet equipped with a flush tank to release a rapid surge of water when a button is pressed. In the future, we will extend the analysis to other types of elements, such as different types of shower heads and toilet flushing. All the analyzed bathrooms had tiled walls up to approximately 180 cm in height, one glass shower enclosure and one masonry shower enclosure, tiled floors, and a window. The characteristics of the bathrooms are as follows:

- Bathroom S1: 185 × 200 h270 with washbasin, bidet and toilet
- Bathroom M1: 3000 × 200 h270 with washbasin, shower tray, bidet and toilet
- Bathroom S2: 240 × 210 h270 with washbasin, shower tray, bidet and toilet
- Bathroom M2: 4800 × 185 h270 with washbasin, bidet and toilet

**Figure 4:**
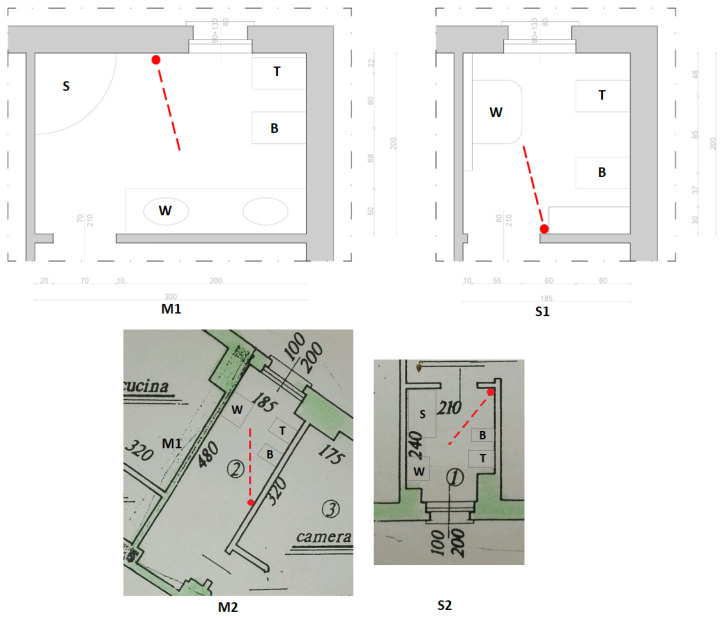
Bathroom layouts for apartments 1 and 2, both including a small (S1, S2) and a medium-sized (M1, M2) bathroom. For example, S1 denotes the small bathroom in apartment 1. Fixtures are labelled as follows: S = shower, T = toilet, W = washbasin, B = bidet. Red points and dashed lines indicate the positions and orientations of the microphones.

Their layouts and positions of the microphones are depicted in Figure 4.

### 4.2. Data Acquisition System

**Figure 5:**
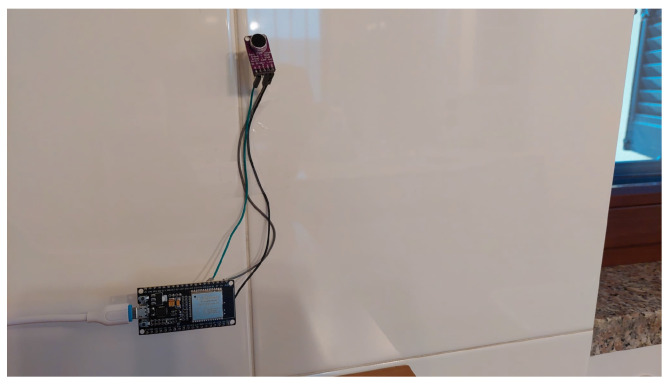
Setup of the microphone and ESP32 in bathroom M1.

The data are acquired using a microphone (Electret Microphone Amplifier MAX9814, Adafruit Industries LLC, New York, NY, USA) with a bandwidth of 20–20,000 Hz, equipped with an automatic gain control (AGC) amplifier. The amplifier’s output is 2 V_pp_ at a 1.25 V DC bias, making it compatible with any analogue-to-digital converter accepting up to 3.3 V input. The microcontroller used is an ESP32 (Espressif Systems, Shanghai, China) with built-in Wi-Fi and Bluetooth. Figure 5 shows an example of a setup with a microphone and ESP32.

The data are sampled at a frequency of 11,200 Hz, which, according to the Nyquist theorem, corresponds to an audio bandwidth of 5600 Hz. A Fast Fourier Transform (FFT) on 1024 samples is performed directly on the microcontroller. Each FFT processes 1024 samples, generating 1024 complex coefficients; however, only the first 512 coefficients (from 0 to 511) represent unique frequencies ranging from 0 Hz (DC component) up to the Nyquist frequency. The coefficients from 512 to 1023 are redundant, as they correspond to the conjugate reflection of the first 511 coefficients. Only these first 512 frequency bins have physical meaning and are used in the subsequent analysis. Each value of the vector is the magnitude, calculated using both the real and imaginary parts. The signal spans from 0 to 5600 Hz with a resolution of approximately 11 Hz. The sampling period is T = 182 ms, so each 1-second window contains just over 5 FFTs.

All acquired data are sent over Wi-Fi to a remote server for processing/storage.

The FFT time sequence was used to create spectrograms with the purpose of visually evaluating the observed phenomena. Each FFT vector was then labelled (ground truth) to generate the input vectors used for training the AI model.

### 4.3. Visual Analysis

The acquired FFTs were used to generate spectrograms for a visual analysis of the data. In particular, the objective was to determine whether each water-related element (washbasin, bidet, toilet, and shower) produces a distinctive acoustic pattern. Establishing the presence of such patterns is a preliminary step to assessing the feasibility of classifying events using a neural network-based approach.

Data were collected from the bathrooms shown in Figure 4 and all measurements were conducted with the door closed.

The spectrograms presented below consist of visualizations of the matrix with dimensions [506, 100]. Each spectrogram represents 100 consecutive FFTs—one per column—each comprising 506 frequency bins. The x-axis covers approximately 18.2 s (100 × 182 ms), while the y-axis spans the frequency range from 100 Hz to 5600 Hz, with a frequency resolution of about 11 Hz per bin.

**Figure 6:**
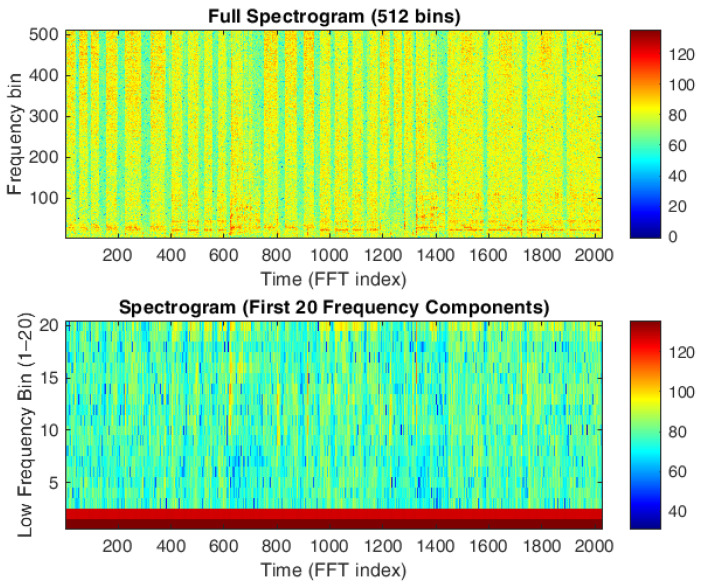
Example of a full spectrogram (**top**) and a spectrogram with only the first 20 frequency components (**bottom**) for different, individually activated water sources in bathroom M1. The top panel shows the complete frequency content, while the bottom panel emphasizes the low-frequency components most relevant for distinguishing taps, shower, and bidet.

To enhance the resolution of the color bar in the frequency ranges of interest, the first two frequency bins (i.e., DC and 11 Hz frequency) were deliberately excluded, based on the observation that these two component contributions do not provide information for distinguishing between different water-consuming sources (see Figure 6). It is important to note that this assumption has also been validated numerically, showing that only the first two components actually lacked significant content. The color bar values are plotted on a logarithmic scale (not in decibels, but in natural logarithmic units, multiplied by 10) to improve spectrogram readability.

**Figure 7:**
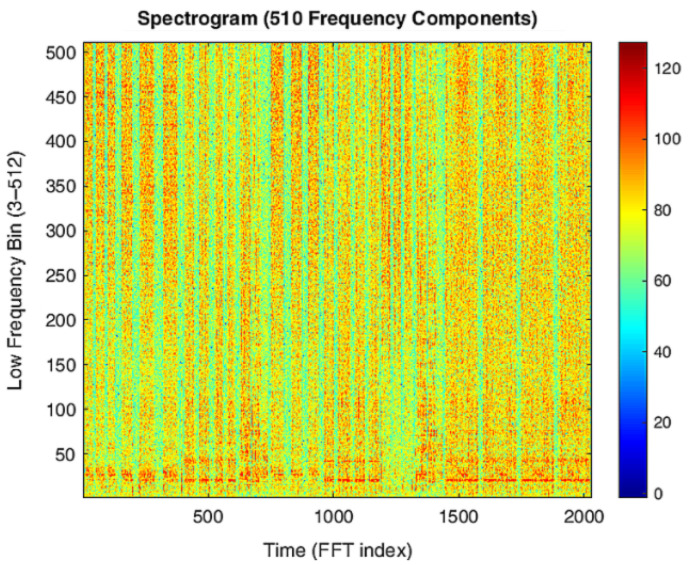
Spectrogram of a series of recordings alternating periods of water usage and inactivity.

Figure 7 shows the spectrogram of a series of recordings with the first two frequency components (DC and 11 Hz) removed. The spectrograms in Figure 6 and Figure 7 clearly differentiate between periods of water usage and inactivity, as indicated by distinct colored bands. This qualitative evidence provides a strong motivation to further investigate the possibility of identifying specific usage patterns and to develop reliable classification models based on these differences.

**Figure 8:**
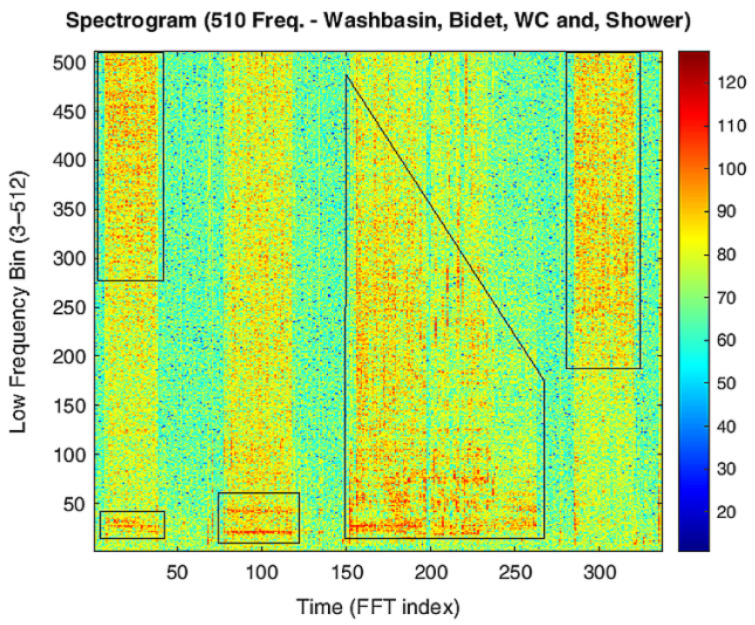
Spectrogram of individual water source activations in bathroom M1. The highlighted regions, from left to right, correspond to the usage of the washbasin, bidet, toilet, and shower. Each region displays the frequency and temporal patterns produced by the respective fixture, illustrating how the acoustic signatures differ between sources and can be used for accurate classification.

Figure 8 presents individual spectrograms corresponding to each water-consuming source, with only one source activated at a time. Each spectrogram provides a representative example of the characteristic acoustic signature associated with a specific type of usage. It is worth noting that there is no complete overlap among the spectrograms, and the observed differences appear to characterize each water source distinctly. This suggests the potential for reliable discrimination between usage types based on their acoustic profiles.

Two phenomena can be observed. First, temporal dependencies within each FFT component suggest that temporal convolution may be beneficial—and possibly necessary in the case of the WC—for capturing sequential patterns over time. Second, the presence of noise and oscillatory behavior across adjacent frequency bins indicates that applying a moving average (MA) filter along the frequency axis could help attenuate high-frequency fluctuations and enhance feature stability.

As previously discussed, the acoustic signatures of individual water-consuming sources exhibit distinguishing characteristics. Consequently, when multiple sources are activated simultaneously, the resulting signal s[n] can be modelled as the linear sum of the individual signals:

(4) $$s\left[n\right]=x_{1}\left[n\right]+x_{2}\left[n\right]+\cdots +x_{k}\left[n\right],$$

where $x_{i}\left[n\right]$ denotes the signal generated by the *i*-th source. Given the linearity of the Discrete Fourier Transform (DFT), and hence the FFT, the spectrum of the combined signal is given by:

(5) $$S\left[k\right]=\mathrm{FFT}\{s\left[n\right]\}=\sum_{i=1}^{k}\mathrm{FFT}\{x_{i}\left[n\right]\}.$$

This means that the frequency components of the overall signal are simply the sum of the corresponding frequency components of the individual source signals.

Therefore, even when multiple sources are active at the same time, their spectral contributions remain superimposed but individually preserved. This property suggests that the composite FFT should still contain sufficient information to enable the identification of each active source, thereby reinforcing the validity of spectral analysis as a means for multi-source recognition.

**Figure 9:**
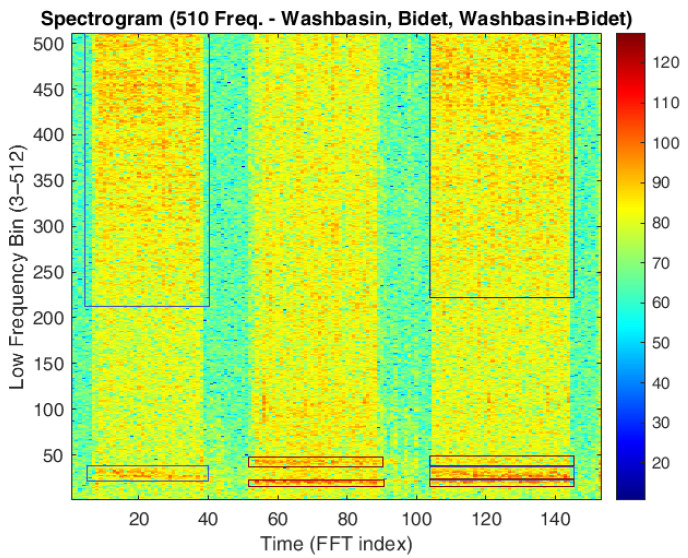
Spectrograms showing, from left to right, the washbasin, the bidet, and their simultaneous activation. The visualization highlights the distinct frequency and temporal patterns of each source and illustrates how overlapping activations produce combined acoustic signatures, which can be used to distinguish and classify multiple water sources.

Figure 9 shows an example of the concurrent activation of the washbasin and bidet. Although the distinction is not highly evident—mainly due to the limited resolution of the spectrogram—it is nevertheless possible to visually identify the spectral contributions of each source during simultaneous operation.

### 4.4. Neural Network Architecture Design

To identify a suitable neural network architecture with 510 input features and 5 output classes, several configurations were tested with the goal of achieving a lightweight, computationally efficient model suitable for embedded systems, while avoiding both underfitting and overfitting.

The description of the vectors used in this evaluation, including their derivation, transformation, and use, will be thoroughly detailed in Section 4.5.

Specifically, three different 3-layer architectures were evaluated:

- 506–256–128–5
- 506–128–64–5
- 506–100–20–5

as well as a deeper 4-layer network:

- 506–256–128–64–5

All architectures employed ReLU activation functions in the hidden layers and a sigmoid activation in the output layer. For each configuration, 10 independent training–validation–testing sessions were conducted. The training process was run for 1000 epochs with a batch size of 32 and a learning rate of 0.0001. The training was performed on 40% of the full annotated dataset (2788 labelled vectors), randomly sampled. Validation was carried out on the remaining training vectors, while a fixed test set of 2020 vectors was used for final performance evaluation.

Results across the 3-layer networks showed no significant differences. In particular, the training loss consistently remained below 0.00005, the validation loss at convergence was below approximately 0.1, and the test loss remained under 1.5. Binary Cross Entropy (BCE) loss was used throughout: summed for training, and without reduction for validation and testing.

The 4-layer network performed slightly worse in terms of generalization, though not significantly.

Given the intention to deploy the model on tiny computing architectures, the best tradeoff between performance and efficiency was achieved by the 506–100–20–5 configuration. It exhibited no signs of underfitting and was computationally faster than the other tested architectures.

### 4.5. Data Processing and Classification

#### 4.5.1. Data Collection

**Figure 10:**
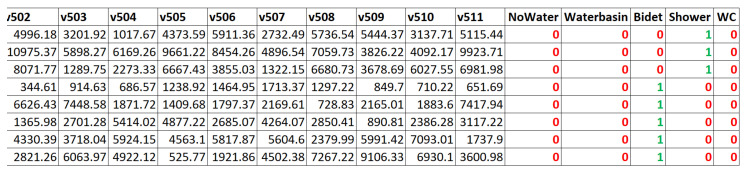
Excerpt of some of the vectors used. In particular, some of the final frequency components are shown, along with the source each vector refers to (1 in green).

The input vectors consist of 512 components, obtained from a 512-point Fast Fourier Transform (FFT) with frequency bins spaced at 11 Hz. An additional 5-bit encoding is appended: one bit indicates that no fixture is in use, while the remaining four represent the washbasin, bidet, shower, and toilet, respectively. These four bits can be activated simultaneously to reflect the concurrent use of multiple water sources. Figure 10 illustrates a subset of sample vectors employed during the identification, validation, and testing phases of the model, showing only the final components of the FFT. The vectors are derived directly from the sampling and FFT pipeline. For both training and validation, only vectors associated with unambiguous ground truth conditions were retained. In all cases where labelling uncertainty was possible, the corresponding vectors were discarded to ensure data quality and consistency. All vectors are timestamped and were manually annotated.

#### 4.5.2. FFT Component Selection

**Figure 11:**
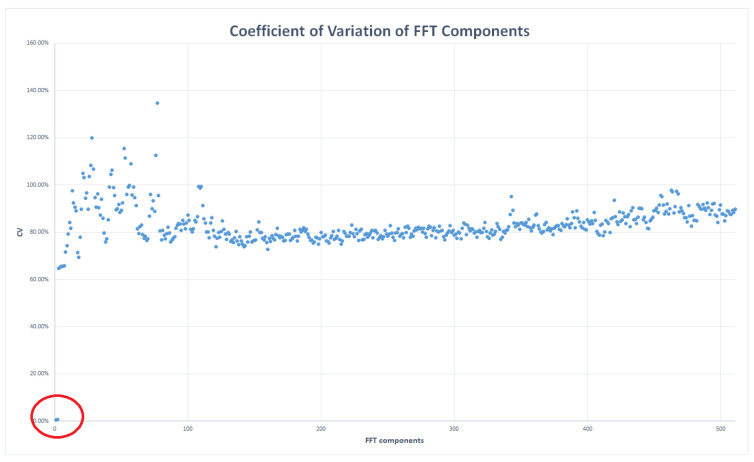
Coefficient of Variation (CV) values computed for all vectors. The first two components, circled in red, display the lowest variability.

In order to determine whether all components carry informative content, an analysis was performed on all collected vectors. The results show that the first two components, continuous (denoted as V0) and 11Hz (denoted as V1), do not contain informative content and were therefore discarded. As indicated in the methodology (Section 3.1), we employed the coefficient of variation (CV), defined as the ratio of the standard deviation to the mean for each bin; it is worth noting that the CV quantifies the relative dispersion of the data with respect to the mean value. Specifically, we obtained CV values of 0.54% and 0.73% for V0 and V1, respectively (Figure 11—values highlighted with a red circle); this indicates that the data series is highly stable and does not carry informative content. The remaining 510 components exhibited CVs exceeding 64.8%, with a mean CV of 84.3% (Figure 11).

#### 4.5.3. Noise Mitigation Along Frequency Dimension

**Figure 12:**
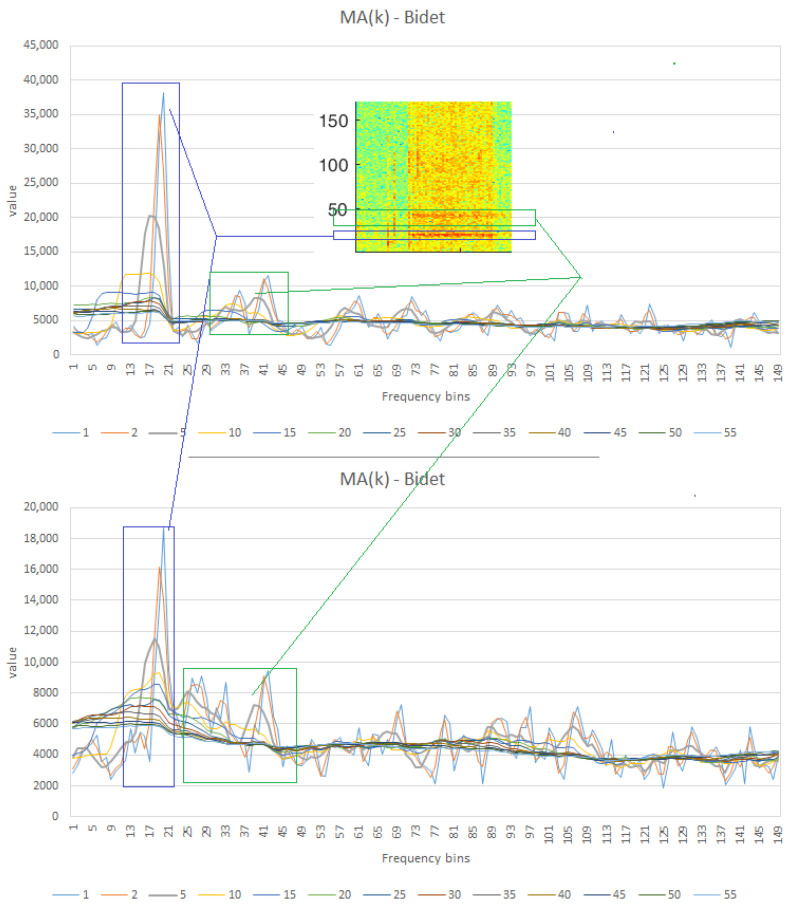
Example of the Moving Average (MA) applied to two bidet signal samples, each consisting of three values. The different curves illustrate the effect of various window sizes k on signal smoothing: smaller values preserve sharp transitions but are more sensitive to noise, whereas larger values reduce noise but may also smooth out distinguishing features. The selected window size for the MA was k = 5. Blue and green shaded regions indicate the corresponding segments within the spectrogram, showing how MA stabilizes the frequency components used for classification.

The visual observation reported in Section 4.3 highlights that the water consumption sources are distinctively characterized (0 corresponds to an inactive water source, 1 to an active water source).For example, the bidet exhibits two well-defined horizontal bands, while the washbasin shows a low-frequency band accompanied by additional components at higher frequencies. To better highlight these phenomena and attenuate variations due to noise (both environmental and sampling), a moving average of order 5 (MA(5)) was applied along the frequency dimension. The value was calculated by analyzing moving averages (MA) with window sizes ranging from 5 to 50 values, with increments of 5, for both the bidet and the washbasin, randomly selecting contiguous windows of 5 values along the temporal dimension. Figure 12 illustrates the effect of the moving average MA(K): values of K≥5 are prone to noise that could affect classification, whereas higher values (K≥15) reduce the distinguishing features. The selected window size for the moving average was K=5.

It is worth noting that convolution along the frequency dimension reduces bin-to-bin variability, thereby strengthening statistical analysis and improving the stability of spectral pattern recognition. Moreover, this process helps tolerate small sampling frequency jitters—on the order of a few Hz—which, in practice, facilitates deployment across different microcontroller platforms, where less stable clocks affect both the ADC section and computation.

#### 4.5.4. Noise Mitigation Along Time Dimension

As in the analysis along the frequency dimension, an investigation was conducted considering different scenarios. Increasing the moving average window size progressively attenuates noise, but at the cost of increased delay in recognition. Instead, with respect to the use of computing architectures with limited resources (e.g., Xtensa LX6, ARM Cortex-M4, ARM Cortex-M7, or STM32H7), no significant issues were observed.

The implementation recommends the use of circular arrays. A circular array is a linear data structure used to efficiently manage sequences of elements with a fixed-size buffer, allowing overwriting of the oldest data once the buffer capacity is reached. In software implementation, the circular array employs a pointer or index that increments with each insertion and, upon reaching the end of the array, wraps around to the beginning, thus forming a logical “ring.” This structure enables maintaining a sliding window of data, as in the case of moving averages, reducing computational complexity and avoiding element shifting operations. Access to elements is managed via modulo operations on the array size, ensuring constant-time insertion and reading. It is worth noting that this approach is particularly suitable for embedded systems with limited resources, where efficient memory management and reduced computational time are critical.

For window sizes of 4, 8, 16, 32, and 64 elements, no significant benefits were observed beyond 16 elements. Therefore, a window size of K = 8 was selected, a good tradeoff between area and noise reduction.

## 5. Normalization MinMax

The relative position of water consumption sources with respect to the microphone affects the FFT values. Consequently, it is important to normalize the input or to apply techniques that make the model invariant to signal power; otherwise, the classification may be influenced more by the signal intensity than by its actual content. Moreover, normalization to the [0, 1] range is also advantageous for neural modelling. It accelerates training convergence, improves numerical stability in dense networks during computations, and helps keep weights and biases balanced. In addition, activation functions operate more effectively when inputs are within a limited range, reducing saturation effects that slow down learning. Finally, normalization promotes better generalization of the model to new data, minimizing the risk that some features dominate excessively.

Each feature vector *j* is individually normalized by computing the minimum and maximum values across its elements, and transforming each value as:

(6) $${\mathrm{NormalizedVal}}_{i,j}=\frac{val_{i,j}-\min_{j}}{\max_{j}-\min_{j}}$$

This normalized vector is used as input to the classification model during the operational phase of the network. Normalized vectors are also used during the training, validation, and testing phases.

## 6. Vectors and Class Balancing

The vectors were extracted from different segments of recordings taken in the four bathrooms. The data are approximately evenly distributed between the two bathrooms with a shower and the two without. Each vector is associated with a timestamp, allowing the identification of the performed activity. The vectors used for training and testing were manually labelled. To avoid ambiguous cases—mainly due to the difficulty of aligning manually recorded times with automatically logged data—transitional phases or segments with uncertain classification were excluded. Specifically, we annotated the start and duration of each event and then selected the central portion of the corresponding data to ensure a reliable alignment between the label and the actual activity. Consequently, only the central vectors were considered for each experiment, accounting for slightly more than 40% of all recorded samples.

Given a sampling interval of 128 ms (approximately 8 samples per second), the total duration of valid recordings amounts to approximately 136 min. Of these, 93 min were used, resulting in a total of 38,035 vectors. This reduction is due to the need for class balancing: in particular, 43% of the valid data belong to the *NoUse* class. The smallest class is *Toilet*, representing 3.7% of the data, while the other classes are around 6%. The size of the *NoUse* class was therefore significantly reduced to achieve a balanced class distribution, bringing it in line with the sizes of the other activity classes.

Out of the 38,035 vectors, 10,115 were used for testing, while the remaining 27,920 were used for training and validation. Of these, 40% (11,168 vectors) were randomly selected for training.

Although there is no dataset size that universally guarantees sufficiency, certain methods can be applied to evaluate it. In our case, we employed a learning curve analysis, relating the loss to the size of the training set. The curve was generated by progressively increasing the portion of the dataset used for training, from 10% up to 80%. For each percentage level, ten independent runs were performed, and the mean and variance of the resulting losses were calculated (Table 1).

The learning curve shows a rapid decrease in loss as the training set grows, followed by a plateau from approximately 35% onward. This stabilization indicates that 40% of the available dataset is sufficient to train the model effectively, as adding more data yields only marginal performance improvements.

It is important to note that these vectors represent a superset of those used in the network size analysis (Section 4.4). In the earlier phase, 2788 vectors were employed as a diverse subset of the full set of 27,920 vectors. This reduced set was more than sufficient to explore and understand the impact of model complexity. The fact that the current experiments are based on an extended dataset does not compromise the validity of the results, as the subset used for network design was representative and well-balanced [21].

## 7. Training and Validation

The 506–100–20–5 network, using ReLU activation functions in the hidden layers and a sigmoid function at the output layer, was trained with a batch size of 32, for 1000 epochs, and a learning rate of 0.0001. The training was performed in the PyCharm (PyCharm 2023.3.4) environment, using Python (Python 3.11) and the Torch (Torch 2.7.0) library.

**Figure 13:**
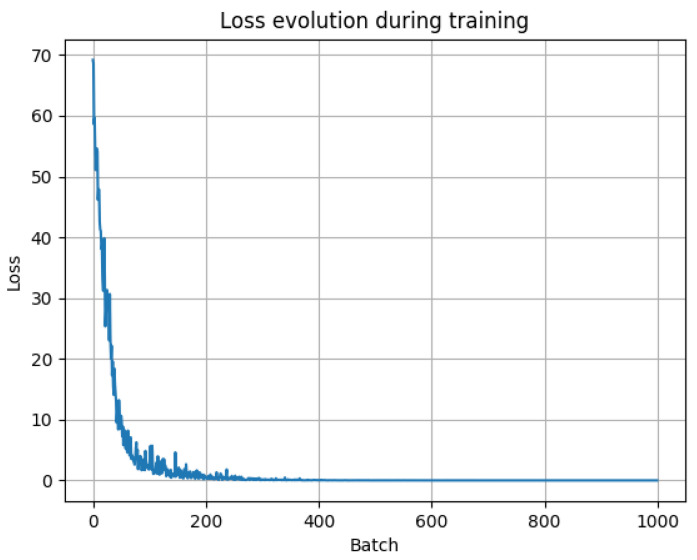
Loss evolution during training.

The result of the training loss function is shown in Figure 13.

## 8. Test

The test was conducted on 10,115 vectors. The results are reported in Table 2 and Table 3. The overall system accuracy is 90.6%.

The confusion matrix indicates overall good system performance. The test phase was conducted by applying the complete methodology that includes moving averages in both the frequency and time domains. The averaging of the active source labels (1 for active, 0 otherwise) was also computed. This step is necessary only during the testing phase, as the activation of a water source occurs at a specific moment in time, but its effect spans multiple vectors due to the application of convolution. In this way, the network’s response can be more appropriately evaluated. It is important to emphasize that the averaged label values do not carry any physical meaning; instead, they should be interpreted as follows: for instance, if eight consecutive test vectors are analyzed, some may indicate that certain sources are active, while others may suggest the presence of different active sources. The purpose of the test is exclusively to evaluate the robustness of the proposed methodology and the impact of the moving average process.

**Figure 14:**
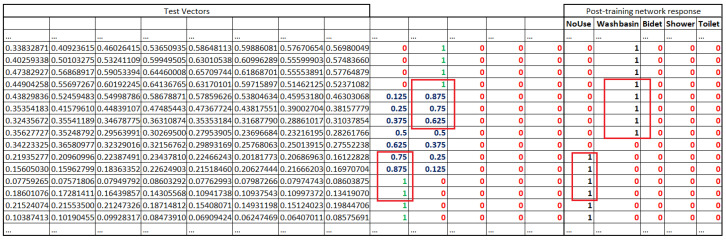
Excerpt from the test during a transient phase from Washbasin to NoUse. Segment of test vectors showing a transition from Washbasin to NoUse. The 8-vector moving average is applied to labels; e.g., 0.125 (1/8) indicates only 1 of 8 vectors was active. A brief classifier indecision (185 ms), in Post-training columnd, is visible as zeros between Washbasin and NoUse. Red values represent 0, blue and green indicate the moving averages of the vectors used for testing, and black values in the Post-training columns correspond to the outputs produced by the network.

Figure 14 illustrates a segment of the test vectors in which a transition from Washbasin to NoUse is observable. The moving average over eight vectors is also applied to the label values: for example, a value of 0.125—which corresponds to 1/8—indicates that only 1 out of 8 vectors was labelled as active. Notice the presence of a brief moment of classifier indecision, lasting approximately 185 ms, during which the output consists entirely of zeros.

## 9. Test with a Synthetic Dataset

Given the impracticality of conducting a large number of real-world experiments, a synthetic dataset was generated. The rationale relies on the linearity property of the Fast Fourier Transform (FFT), which enables the construction of new data through linear combinations of existing vectors. This approach allows not only for the simulation of various noise levels—by increasing uncertainty in the FFT amplitudes—but also for the modelling of signals originating from different environments or varying distances between sound sources and the microphone. For instance, increasing the distance from *d* to 2d reduces the sound pressure amplitude by half, since it decreases proportionally to 1/d. Consequently, the sound intensity—defined as the power per unit area—drops by a factor of four, due to its dependence on the square of the pressure ($1/d^{2}$). From a spectral perspective, this results in a uniform attenuation across all FFT components without modifying their frequency distribution.

It is worth noting that we did not alter the frequency distribution itself, as we lacked the opportunity to experiment with different materials or water fixtures beyond those available in the original setup.

### 9.1. Random Combination

A preliminary test for identifying sources of water consumption was conducted by combining vectors associated with the *washbasin*, *bidet*, and *shower*, selected randomly from the available dataset. The *toilet* class was intentionally excluded, as its operation is characterized by a specific temporal sequence of vectors, and a random combination would distort its temporal pattern.

A total of 11,197 synthetic sequences were generated, with the following class distribution: *shower* (103), *washbasin* (118), *bidet* (190), *shower* + *bidet* (1745), *bidet* + *washbasin* (2692), *shower* + *washbasin* (1644), and *shower* + *bidet* + *washbasin* (5505). These classes were concatenated in sequence and processed according to the proposed methodology, including moving average operations in both the frequency and time domains.

Class labels were smoothed over time using the same moving average process applied to the data vectors. This was necessary because, although the activation of a fixture occurs at a specific time instant, its influence spans multiple vectors that may also capture portions of other overlapping phenomena. Of course, this temporal averaging is not used during real-time operation, where the objective is to detect the active source(s) at any given moment. The goal of this test is solely to assess the robustness of the methodology and the effect of the moving average.

The results are highly encouraging: of the initial 11,197 input vectors, 11,190 remained after preprocessing, and 11,502 classifications were correct. The remaining discrepancies are attributed to transient states—specifically, seven misclassifications for each of the following six transitions:

- bidet → washbasin,
- washbasin → washbasin + bidet,
- washbasin + bidet → shower,
- shower → shower + bidet,
- shower + bidet → shower + washbasin,
- shower + washbasin → shower + washbasin + bidet.

Additionally, 446 misclassifications occurred where the model output values were below the decision threshold of 1. Of these, 384 (86%) involved the configuration where all three sources—washbasin, bidet, and shower—were active simultaneously (a class comprising 5498 vectors or 6.9% of the dataset). These misclassifications typically occurred as single-vector spikes (128 ms duration), with the longest erroneous sequence spanning 9 consecutive vectors (just over one second).

By applying a stricter output threshold of >0.5, the number of incorrect classifications drops to 155, yielding an error rate of 155/11,190 × 100 = 1.38%, and an overall classification accuracy of 98.6%.

### 9.2. Dominant Source and Added Noise

To simulate more realistic and complex acoustic scenarios, tests were conducted in which a single source is considered dominant while the others are attenuated. This type of simulation is meaningful only in the presence of two or more simultaneously active sources, as MinMax normalization applied to each vector cancels the attenuation effect in the case of a single active source.

The general model applied to each test vector is as follows:

(7) $$FFT_{i\text{-}new}=FFT_{i}\cdot \alpha +\beta ,\;\beta ∼\mathrm{WN}\left(0,\sigma^{2}\right)$$

where β represents a white noise process with zero mean and variance $\sigma^{2}$, used to model ambient noise added to the spectral components. The parameter α∈[0,1] is an attenuation factor related to the source distance: for instance, α=0.5 simulates a doubling of the distance, assuming an amplitude decay proportional to the inverse of the distance. It is worth noting that each component $FFT_{i}$ is uniformly scaled by α, as in free-field conditions, sound pressure decays with 1/d, and the FFT is a linear transformation. Thus, reducing signal amplitude simulates increased distance across the entire frequency spectrum.

Regarding β, since noise is not constant but depends on the context—such as the reflective properties of materials, and the presence of other sounds and environmental noise—and since perceived noise tends to increase proportionally to the intensity of the useful signal, a reasonable model for σ is the following:

(8) $$\sigma =\gamma \cdot \sigma ({\mathrm{FFT}}_{i})$$

where γ is a parameter that controls the noise level. Empirically, and for applications in audio and signal processing, the reference is often the Signal-to-Noise Ratio (SNR).

Note that

(9) $${\mathrm{SNR}}_{\mathrm{linear}}=\frac{\sigma_{\mathrm{signal}}^{2}}{\sigma_{\mathrm{noise}}^{2}}$$

with $\sigma_{\mathrm{noise}}=\gamma \cdot \sigma_{\mathrm{signal}},$

$${\mathrm{SNR}}_{\mathrm{linear}}=\frac{\sigma_{\mathrm{signal}}^{2}}{{\left(\gamma \;\sigma_{\mathrm{signal}}\right)}^{2}}=\frac{1}{\gamma^{2}}.$$

Converting to decibels:

(10) $${\mathrm{SNR}}_{dB}=10\log_{10}\left(\frac{1}{\gamma^{2}}\right)=-20\log_{10}(\gamma ).$$

this yields

(11) $$\gamma =10^{-\frac{{\mathrm{SNR}}_{dB}}{20}}.$$

Indicatively, an SNR of approximately 20–30 dB is considered a low-noise environment of good quality (γ≈0.03−0.1). An SNR around 5–20 dB indicates a noisy environment (γ≈0.1−0.6)—an SNR near 10 dB corresponds to a moderately noisy environment (γ≈0.3)—while an SNR below 5 dB is characteristic of highly noisy conditions (γ≥0.6).

The values of $\sigma ({\mathrm{FFT}}_{i})$ are computed for the individual sources by considering all the experiments carried out. This approximation is reasonable in view of the adopted model. It is worth highlighting that the model allows specifying a γ for each FFT frequency component, thus enabling the simulation of colored noise (for example, pink noise of the form $\gamma (f)=\frac{\gamma }{f^{\alpha }}$ with α=1; note that α=0 yields white noise).

The number of vectors used amounts to 2023, randomly selected and modified or combined according to the specific experiment performed. In particular, the experiments include: single-source noisy environment, multi-source noisy environment, and multi-source scenarios where the distance of one source was varied.

**Figure 15:**
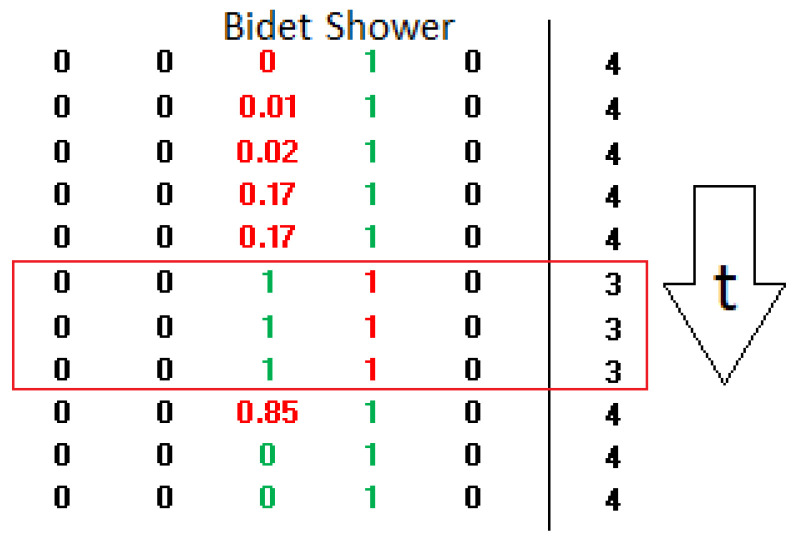
Flow of source classifications.

It is worth noting that the following is a static analysis, where performance is measured by comparing the system’s output at each time t to the corresponding reference. Conversely, considering the temporal sequence of vectors would likely lead to further improvements in performance. This approach requires the adoption of post-classification strategies, such as median filtering or the application of hysteresis mechanisms. For instance, in Figure 15, the brief period of indecision—during which the output alternates between bidet and shower—could be consistently resolved in favor of shower, since it is improbable that the water would be turned off and immediately on again within such a short time frame.

### 9.3. Ambient Noise

In this simulation, the environmental complexity increased by analyzing the effect of noise on source recognition. This approach made it possible to assess the robustness of the model under more complex and realistic conditions, including different background noise levels, and to highlight potential weaknesses. The goal was to evaluate the system’s ability to discriminate and process signals even in environments characterized by high variability and a higher density of acoustic events. This analysis could also provide useful insights for future work. Below, for the sake of brevity, only the source and the experimental results are reported, computed as the average over 10 runs conducted for each scenario. Specifically, the following metrics are presented: the number of unrecognized vectors (also expressed as a percentage), precision (P), recall (R), and the F1 score. The false negatives correspond to instances where the network fails.

- Single source, Noise model γ = 0.01 (good quality environment)
  – NoUse: FN = 3 (0.1%), P = 100%, R = 99.6% and, F1 = 99.8%, loss = 0.0067.
  – Washbasin: FN = 0 (0.0%), P = 100%, R = 100% and, F1 = 100%, loss = 0.015.
  – Bidet: FN = 17 (8.4%), P = 100%, R= 92.2% and, F1 = 99.6%, loss = 0.05.
  – Shower: FN = 0 (0.0%), P = 100%, R = 100% and, F1 = 100%, loss = 0.0003.
- Single source, Noise model γ = 0.3 (moderately noisy environment)
  – NoUse: FN = 16 (0.8%), P = 100%, R = 99.2% and, F1 = 99.6%, loss = 0.012.
  – Washbasin: FN = 0 (0.0%), P = 100%, R = 100% and, F1 = 100%, loss = 0.029.
  – Bidet: FN = 30 (18.9%), P = 100%, R = 98.5% and, F1 = 98.3%, loss = 0.056.
  – Shower: FN = 3 (1.4%), P = 100%, R = 99.9% and, F1 = 99.9%, loss = 0.001.
- Single source, Noise model γ = 0.8 (highly noisy environment)
  – NoUse: FN = 15 (0.7%), P = 100%, R = 99.3% and, F1 = 99.6%, loss = 0.013.
  – Washbasin: FN = 0 (0.0%), P = 100%, R = 100% and, F1 = 100%, loss = 0.049.
  – Bidet: FN = 24 (1.2%), P = 100%, R = 98.8% and, F1 = 99.4%, loss = 0.137.
  – Shower: FN = 280 (13.8%), P = 100%, R = 86.2% and, F1 = 92.6%, loss = 0.055.

These are examples of additional simulations in which sources were superimposed and environmental noise was introduced. All simulations were designed to be realistic; here, we report the results of one representative configuration among several tested.

- Bidet and Sink
  – Noise level: 0.3, Loss = 0.019. There are 37 vectors for which the washbasin is not recognized, and 4 vectors for which the bidet is not recognized. All non-recognition events occur in bursts with a maximum length of 10 contiguous vectors (approximately 2 s). The shower is consistently recognized.
  – Noise level: 0.8, Loss = 0.019. There are 33 vectors for which the washbasin is not recognized, and 5 vectors for which the bidet is not recognized. All non-recognition events occur in bursts with a maximum length of 5 contiguous vectors (approximately 1 s).
- Bidet and Shower
  – Noise level: 0.3, Loss = 0.048. There are 25 vectors in which the washbasin is recognized (bursts up to 2 vectors) and 4 vectors in which the shower is not recognized.
  – Noise level: 0.8, Loss = 0.032. There are 37 vectors in which the washbasin is recognized (bursts up to 4 vectors) and 9 vectors in which the shower is not recognized.
- Sink, Bidet, and Shower
  – Noise level: 0.3, Loss = 0.065. There are 84 vectors for which the washbasin is not recognized (bursts up to 6 vectors), 6 vectors for which the bidet is not recognized, and 16 vectors for which the shower is not recognized.
  – Noise level: 0.8, Loss = 0.121. There are 133 vectors for which the washbasin is not recognized (bursts up to 10 vectors), 8 vectors for which the bidet is not recognized, and 49 vectors for which the shower is not recognized.

### 9.4. Distance Modification

In this simulation, two or more sources are active simultaneously, with one source virtually moved farther away by doubling or tripling its distance. This setup is used exclusively to assess the robustness of the algorithm.

Note that in the experiments, the distances between each source and the microphone were as follows:

- M1: Shower 1.0 m, Toilet 1.4 m, Bidet 1.5 m, washbasin 1.6 m.
- S1: Toilet 1.5 m, Bidet 1.4 m, Washbasin 0.9 m.
- M2: Toilet 1.7 m, Bidet 1.1 m, Washbasin 2.0 m.
- S2: Shower 1.6 m, Toilet 1.6 m, Bidet 1.1 m, Washbasin 2.3 m.

As the vectors were mixed, the average effective distances are approximately: shower 1.3 m, bidet 1.4 m, washbasin 1.5 m, and toilet 1.6 m. Doubling the distance places the sources beyond 2 m, while tripling moves them beyond 3 m.

The washbasin and the shower at double distance (which could also correspond to a shower structure attenuating its noise) were tested with a noise level of 0.3, obtaining a loss of 0.208. There are 61 vectors correctly recognizing the washbasin and 297 vectors (15%) failing to recognize the shower, with bursts of up to 20 consecutive non-recognitions (approximately 4 s). The increased distance makes source recognition critical. When the shower distance is tripled under the same noise level (0.3), the obtained loss increases to 1.248. In this case, there are still 61 vectors recognizing the washbasin, but 1193 vectors (59%) fail to recognize the shower, with bursts reaching up to 44 consecutive non-recognitions (approximately 9 s). It is evident that the closer source masks the more distant consumption source, making recognition extremely difficult.

An environment with the bidet and the shower positioned at double distance was also evaluated (noise 0.3). The addition of the environmental noise aims to simulate increased source distance. It should be noted that without adding the environment, the min–max normalization would compensate for the reduction in signal amplitude. In this experiment, the loss reached 5.6375. The environmental component itself is not classified because active sources are present, which is exactly the expected behavior. A total of 89 vectors (4%) failed to recognize the washbasin, 221 vectors (11%) failed to recognize the shower, while 196 vectors (10%) correctly recognized the bidet. The longest burst reached 17 consecutive non-recognitions, while all other bursts were shorter than 8.

When the distance increased by a factor of three, while keeping the same active sources, the loss reached 4.613. In this condition, for some vectors (315, 15%), the sources were not detected at all. Specifically, 494 vectors (14%) failed to recognize the washbasin, 865 vectors (43%) failed to recognize the shower, while 159 vectors (18%) correctly recognized the bidet. Regarding the bursts, they varied in length from a few vectors up to more than 20 consecutive non-recognitions. In this simulation, the environmental noise significantly hindered source identification.

## 10. Limitations

The proposed methodology is general, although it is plausible that the model may require updating through a data sampling session followed by data transformation and retraining. Training was conducted in environments with similar characteristics and has demonstrated good performance also in inter-environment settings, i.e., learning in two environments and applying the model to the other two. Although the methodology enables the identification of a model tailored to a specific environment (e.g., a different kitchen or bathroom), the obtained results naturally reflect the properties of the bathrooms used in the experiments. In particular, all bathrooms were tiled up to three quarters of the wall height and were equipped with ceramic sinks, bidets, and shower trays. All water outlets included a diffuser filter (aerator and sand filter). The showerheads were non-adjustable but varied in diameter (22 cm and 10 cm). Each bathroom also featured a window. The toilet flushing system was button-operated, producing a fast but decaying water flow. It should be noted that bathtubs were not included in the experiments, as they have become increasingly rare and are often replaced by showers due to considerations of water efficiency. These characteristics should be considered when assessing the applicability of the results to other contexts.

Future investigations should consider further scenarios, such as kitchens: here, different user activities may introduce different acoustic and hydraulic signatures. Moreover, it should be tested in multi-resident households with overlapping usage events. Finally, tests with non-ceramic bathroom fixtures (e.g., composite or metallic materials) are needed to show the robustness and generalizability of the proposed approach.

## 11. Dimensional Analysis of RAM Requirements and Architecture Selection

To assess the feasibility of implementing the system on tiny architectures, the RAM requirements are estimated for a configuration based on a 1024-point FFT algorithm, a moving average (MA) filter of order 5 along the frequency dimension, and a temporal moving average requiring storage of 10 vectors of 1014 floating-point values each. Additionally, a dense neural network with architecture 1014–100–25–5 is considered. For the 1024-point FFT in floating point, memory usage for input, output, and intermediate buffers is approximately 8 KB, as reported by common DSP libraries. The frequency-domain moving average requires about 4 KB to store a single vector, while the temporal moving average uses around 40 KB. The dense neural network is the main contributor to memory consumption. The first layer (1014 inputs to 100 neurons) requires about 400 KB for weights and biases. The subsequent layers (100–25 and 25–5) need approximately 10 KB and 0.5 KB, respectively, plus about 1 KB for activations.

In total, the estimated RAM requirement is around 460 KB. It is recommended to use microcontrollers with at least 500 KB of free RAM to ensure adequate operating margin, including space for stack, temporary variables, and additional buffers. Suitable platforms include microcontrollers such as the STM32H7 (STMicroelectronics, Geneva, Switzerland) or ESP32 with PSRAM (Espressif Systems, Shanghai, China).

## 12. Concluding Remarks and Future Developments

This paper proposed a lightweight, noninvasive system capable of identifying water flow sources, enabling smart-home-level activity monitoring without plumbing modifications. This is particularly important to support early detection of cognitive decline through changes in bathroom activity patterns.

The focus of this paper was directed toward assessing the practical applicability of the proposed idea, considering important aspects such as robustness to noise, multi-label classification, and efficiency on microcontrollers.

The proposed methodology achieves robust classification of domestic water usage events. The trained network (506–100–20–5) attained an overall accuracy of 90.6% on over 10,000 test vectors, with precision and recall consistently above 87%, confirming its reliability across different classes. Misclassifications mainly occur during short transient phases between usage states. The moving averages along frequency and time dimensions reduce variability and improve recognition stability, enabling deployment on microcontroller platforms with less stable clocks.

Given the impracticality of extensive real-world testing, a synthetic dataset was generated leveraging the linearity of the FFT, allowing the creation of new vectors through linear combinations of existing data. This simulates various noise levels and environmental conditions, including variations in source-to-microphone distance, which uniformly attenuates the spectral components without altering frequency distribution. Frequency characteristics were kept unchanged due to limited variations in materials and fixtures.

A preliminary test on 11,197 synthetic sequences—random combinations of washbasin, bidet, and shower data—showed excellent results, with 98.6% classification accuracy after applying a stricter decision threshold. Most misclassifications were transient and short-lived spikes during transitions or simultaneous activation of all sources.

More complex scenarios were simulated where two or more sources were active simultaneously, with one source virtually moved farther away by doubling or tripling its distance. Distances in the experiments ranged roughly from 0.9 m to 2.3 m, and doubling or tripling these distances placed sources beyond 2 m or 3 m, respectively. Tests with sources at increased distances and added noise demonstrated how increased attenuation and environmental noise significantly degrade recognition performance, with the closer source often masking more distant ones, causing bursts of misclassification lasting several seconds.

Specifically, scenarios with washbasin and shower or bidet and shower at doubled or tripled distances, and moderate noise levels (γ=0.3) showed substantial increases in loss and missed recognitions, highlighting the critical impact of distance and noise on source discrimination. These results confirm the robustness and limitations of the approach in realistic multi-source environments.

The analysis is static, evaluating vector-by-vector classification accuracy. Temporal post-processing techniques, such as median filtering or hysteresis, could further enhance performance by smoothing transient ambiguities and represent a promising direction for future work.

The proposed methodology is general, though the model may require updating through environment-specific data sampling, transformation, and retraining. Training was performed in bathrooms sharing similar characteristics—tiled walls, ceramic fixtures, diffuser filters on water outlets, non-adjustable showerheads of varying diameters, and button-operated toilets producing fast but decaying flows. Bathtubs were not included due to their decreasing prevalence.

These environmental characteristics must be considered when applying the results to other contexts, especially in buildings with different architectural or material settings. Extending data collection and model validation across diverse environments will help improve the generalizability and robustness of the approach. Overall, the method demonstrates strong potential to reliably classify domestic water usage on resource-constrained platforms under varied conditions, with clear avenues for enhancement through temporal processing and expanded environmental modelling.

In particular, we plan to expand the dataset and validation across a broader range of households and environments, integrate temporal post-processing strategies to reduce transient misclassifications, and analyze temporal water usage patterns to support human activity recognition (e.g., ADLs). Moreover, we will investigate the estimation of water consumption volumes based on flow intensities and, critically, evaluate the system in real households to assess long-term robustness, noise resilience, and user acceptance.

## 13. Ethical and Privacy Considerations

The proposed system is designed for noninvasive monitoring of household water consumption, with the long-term goal of supporting activities such as early diagnosis of cognitive decline and assistance with daily living. Data collection respects residents’ privacy: audio is processed locally, and only the information essential for identifying water consumption sources is transferred to the server for subsequent feature extraction. Raw audio files are never stored. Users are fully informed about the purpose of the monitoring, the type of data collected, and how it will be used and stored. Consent procedures are clear, documented, and revocable at any time. Pseudo-anonymization and security measures are implemented to prevent unauthorized access, including encryption of stored data and controlled access rights. Ultimately, ethical considerations encompass the responsible use of the system. Any information derived from monitoring should support well-being and autonomy, avoiding misuse or discriminatory practices. Future deployment in real homes should follow established guidelines for ethical research and smart home monitoring, ensuring that the benefits to health and daily life outweigh potential privacy risks.

## Figures and Tables

**Table 1:** Learning curve results showing mean and variance of the loss for 10–80% of the training set. Ten runs were performed per percentage.

Training Set (%)	Mean Loss	Variance
10%	0.40800	0.09708
15%	0.46650	0.01696
20%	0.16100	0.02119
25%	0.08025	0.00005
30%	0.06850	0.00034
35%	0.01113	0.00007
40%	0.01340	0.00002
45%	0.00233	0.00000
50%	0.00823	0.00000
60%	0.00006	0.00000
70%	0.00032	0.00000

**Table 2:** Confusion matrix (percentages) for the classification of water source activations. Rows correspond to the true labels and columns to the predicted labels.

	NoUse	Washbasin	Bidet	Shower	Toilet
NoUse	21.3%	0.2%	1.0%	0.3%	0.9%
Washbasin	2.6%	22.0%	0.4%	0.0%	0.0%
Bidet	3.6%	0.3%	32.7%	0.0%	0.0%
Shower	0.0%	0.0%	0.0%	4.5%	0.0%
Toilet	0.1%	0.0%	0.0%	0.0%	10.1%

**Table 3:** Performance metrics of the proposed classifier, including true positives, false positives, false negatives, precision, recall, and F1-score for each class.

	TP	FP	FN	Precision	Recall	F1
NoUse NoUse	2150	630	245	77.3%	89.8%	83.1%
NoUse Washbasin	2225	50	305	97.8%	87.9%	92.6%
NoUse Bidet	3310	145	390	95.8%	89.5%	92.5%
NoUse Shower	460	30	0	93.9%	100.0%	96.8%
NoUse Toilet	1020	95	10	91.5%	99.0%	95.1%

## Data Availability

The data presented in this study are available on request from the corresponding author due to privacy restrictions.
